# Towards automated crystallographic structure refinement with *phenix.refine*

**DOI:** 10.1107/S0907444912001308

**Published:** 2012-03-16

**Authors:** Pavel V. Afonine, Ralf W. Grosse-Kunstleve, Nathaniel Echols, Jeffrey J. Headd, Nigel W. Moriarty, Marat Mustyakimov, Thomas C. Terwilliger, Alexandre Urzhumtsev, Peter H. Zwart, Paul D. Adams

**Affiliations:** a Lawrence Berkeley National Laboratory, One Cyclotron Road, MS64R0121, Berkeley, CA 94720, USA; b Los Alamos National Laboratory, M888, Los Alamos, NM 87545, USA; c IGBMC, CNRS–INSERM–UdS, 1 Rue Laurent Fries, BP 10142, 67404 Illkirch, France; d Département de Physique, Faculté des Sciences et des Technologies, Université Henri Poincaré, Nancy 1, BP 239, 54506 Vandoeuvre-lès-Nancy, France; e Department of Bioengineering, University of California Berkeley, Berkeley, CA 94720, USA

**Keywords:** structure refinement, *PHENIX*, joint X-ray/neutron refinement, maximum likelihood, TLS, simulated annealing, subatomic resolution, real-space refinement, twinning, NCS

## Abstract

*phenix.refine* is a program within the *PHENIX* package that supports crystallographic structure refinement against experimental data with a wide range of upper resolution limits using a large repertoire of model parameterizations. It has several automation features and is also highly flexible. Several hundred parameters enable extensive customizations for complex use cases. Multiple user-defined refinement strategies can be applied to specific parts of the model in a single refinement run. An intuitive graphical user interface is available to guide novice users and to assist advanced users in managing refinement projects. X-ray or neutron diffraction data can be used separately or jointly in refinement. *phenix.refine* is tightly integrated into the *PHENIX* suite, where it serves as a critical component in automated model building, final structure refinement, structure validation and deposition to the wwPDB. This paper presents an overview of the major *phenix.refine* features, with extensive literature references for readers interested in more detailed discussions of the methods.

## Abstract 2

*phenix.refine* is a program within the *PHENIX* package that supports crystallographic structure refinement against experimental data with a wide range of upper resolution limits using a large repertoire of model parameterizations. This paper presents an overview of the major *phenix.refine* features, with extensive literature references for readers interested in more detailed discussions of the methods.

## 1. Introduction   

Crystallographic structure refinement is a complex procedure that combines a large number of very diverse steps, where each step may be very complex itself. Each refinement run requires selection of a *model parameterization*, a *refinement target* and an *optimization method*. These decisions are often dictated by the experimental data quality (completeness and resolution) and the current model quality (how complete the model is and the level of error in the atomic parameters). The diversity of data qualities (from ultrahigh to very low resolution) and model qualities (from crude molecular-replacement results to well refined near-final structures) generates the need for a large variety of possible model parameterizations, refinement targets and optimization methods.

Model parameters are variables used to describe the crystal content and its properties. Model parameters can be broken down into two categories: (i) those that describe the atomic model (atomic model parameters), such as atomic coordinates, atomic displacement parameters (ADPs), atomic occupancies and anomalous scattering terms (*f*′ and *f*′′), and (ii) non-atomic model parameters that describe bulk solvent, twinning, crystal anisotropy and so on. The parameters that describe the crystal are combined and expressed through the total model structure factors **F**_model_, which are expected to match the corresponding observed values *F*_obs_ and other experimentally derived data (*e.g.* experimental phase information).

A refinement target is a mathematical function that quantifies the fit of the model parameters (expressed through **F**_model_) and the experimental data (amplitudes, *F*_obs_, or intensities, *I*_obs_, and experimental phases if available). Typically, target functions are defined such that their value decreases as the model improves. This in turn formulates the goal of a crystallographic structure refinement as an optimization problem in which the model parameters are modified in order to achieve the lowest possible value of the target function or, in other words, minimization of the refinement target.

Algorithms to optimize the refinement target range from gradient-driven minimization, simulated-annealing-based methods and grid searches to interactive model building in a graphical environment. These methods vary in speed, scalability, convergence radius and applicability to current model parameters. The type of parameters to be optimized, the number of refinable parameters and the current model quality may all dictate the choice of optimization (target-minimization) method.

Below, we describe how crystallographic structure refinement is implemented in *phenix.refine*.

## 2. Methods   

Crystallographic structure refinement can be performed in *PHENIX* (Adams *et al.*, 2002 ▶, 2010 ▶) using X-ray data, neutron data or both types of data simultaneously. Highly customized refinement strategies are available for a broad range of experimental data resolutions from ultrahigh resolution, where an interatomic scatterer (IAS) model can be used to model bonding features (Afonine *et al.*, 2004 ▶, 2007 ▶), to low resolution, where the use of torsion-angle parameterization (Rice & Brünger, 1994 ▶; Grosse-Kunstleve *et al.*, 2009 ▶) and specific restraints for coordinates [reference-model, secondary-structure, noncrystallographic symmetry (NCS) and Ramachandran plot restraints] may be essential (Headd *et al.*, 2012 ▶). A highly optimized automatic rigid-body refinement protocol (Afonine *et al.*, 2009 ▶) is available to facilitate initial stages of refinement when the starting model may contain large errors or as the only option at very low resolution. Most refinement strategies can be combined with each other and applied to any selected part of the structure. Specific tools are available for refinement using neutron data, such as automatic detection, building and refinement of exchangeable H/D sites and difference electron-density map-based building of D atoms for water molecules (Afonine, Mustyakimov *et al.*, 2010 ▶). Most of the refinement strategies available for refinement against X-­ray data are also available for refinement using neutron data. Refinement of individual coordinates can be performed in real or reciprocal space or consecutively in both (dual-space refinement). Refinement against data collected from twinned crystals is also possible.

The high degree of flexibility and extensive functionality of *phenix.refine* has been made possible by modern software-development approaches. These approaches include the use of object-oriented languages, where the convenience of scripting and ease of use in Python are augmented by the speed of C++, and by a library-based development approach, where each of the major building blocks is implemented as a reusable set of modules. Most of the modules are available through the open-source CCTBX libraries (Grosse-Kunstleve & Adams, 2002 ▶; Grosse-Kunstleve *et al.*, 2002 ▶). An overview of the underlying open-source libraries can be found in a series of recent *IUCr Computing Commission Newsletter* articles (issues 1–8; http://www.iucr.org/iucr-top/comm/ccom/newsletters/).

The refinement protocol implemented in *phenix.refine* (Afonine *et al.*, 2005*b* ▶) consists of three main parts.

*Initialization*: includes processing of input data and the job-control parameters, analysis and refinement-strategy selection and a number of consistency checks.
*Macro-cycle*: the main body of refinement, a repeatable block where the actual model refinement occurs.
*Output*: the concluding step where the refined model, electron-density maps and many statistics are reported.

The following sections outline the key steps of structure refinement in *phenix.refine*.

### 2.1. Initial step of refinement: processing of inputs   

To initiate refinement, a number of major sources of information have to be processed.

(i) Structural model: coordinates, displacement parameters, occupancies, atom types, *f*′ and *f*′′ for anomalous scatterers (if present).
(ii) Reflection data: pre-processed observed intensities or amplitudes of structure factors and, optionally, experimental phases.
(iii) Parameters determining the refinement protocol.
(iv) Empirical geometry restraints: bond lengths, bond angles, dihedral angles, chiralities and planarities (Engh & Huber, 1991 ▶; Grosse-Kunstleve, Afonine *et al.*, 2004 ▶).
(v) Optionally, a restraint library file (CIF) may be provided to define the stereochemistry of entities in the input model (for example, ligands) that do not have corresponding restraints in the library included in the *PHENIX* distribution.

The user provides the structural model and reflection data. The refinement software then retrieves default parameters and information from a library of empirical geometry restraints, which can be readily customized by the user.

The PDB format (Bernstein *et al.*, 1977 ▶; Berman *et al.*, 2000 ▶) is the most commonly used format for exchanging macromolecular model data and is therefore available as the input format for refinement in *PHENIX*. The iotbx.pdb library module (Grosse-Kunstleve & Adams, 2010 ▶) performs the first stage of the PDB-file interpretation. It robustly constructs an internal hierarchy of *models* (PDB MODEL keyword), *chains*, *conformers* (PDB altLoc identifier), *residues* and *atoms*. Common simple formatting problems are corrected on the fly where possible. Currently, *phenix.refine* can only make use of PDB files containing a single model. The second stage of the PDB interpretation involves matching the structural data with definitions in the CCP4 Monomer Library (Vagin & Murshudov, 2004 ▶; Vagin *et al.*, 2004 ▶) in order to derive geometry restraints, scattering types and nonbonded energy types. Many common simple formatting and naming problems are considered in this interpretation. The PDB interpretation (iotbx.pdb) has been tested with all files found in the PDB database (http://www.pdb.org/) as of August, 2011 and supports both PDB version 2.3 and version 3.*x* atom-naming conventions. The vast majority of files can be processed without user intervention. Detailed diagnostic messages help the user to quickly identify idiosyncrasies in the PDB file that cannot be automatically corrected. If the input PDB file contains an item undefined in the CCP4 Monomer Library, a geometry restraint (CIF) file must be provided for that item. This file can be obtained by running *phenix.elbow* (Moriarty *et al.*, 2009 ▶) or *phenix.ready_set*, which is more comprehensive and automated.

The experimental data can be provided in many commonly used formats. Multiple input files can be given simultaneously, *e.g.* a *SCALEPACK* file (Otwinowski & Minor, 1997 ▶) with observed intensities, a *CNS* (Brünger *et al.*, 1998 ▶) file with *R*_free_ flags (Brünger, 1992 ▶, 1993 ▶) and an MTZ file (Winn *et al.*, 2011 ▶) with phase information. A comprehensive procedure aims to extract the data most suitable for refinement without user intervention. A preliminary crystallographic data analysis is performed in order to detect and ignore potential reflection outliers (Read, 1999 ▶). If twinning (for a review, see Parsons, 2003 ▶; Helliwell, 2008 ▶) is suspected, a user can run *phenix.xtriage* (Zwart *et al.*, 2005 ▶) to obtain a twin-law operator to be used by the twin-refinement target in *phenix.refine*.

A number of automatic adjustments to the refinement strategy are considered at this point. These adjustments include automatic choice of refinement target if necessary (based on the number of test reflections, the presence of twinning and the availability of experimental phase information as Hendrickson–Lattman coefficients; Hendrickson & Lattman, 1970 ▶), specifying the atomic displacement parameters (isotropic or anisotropic), determining whether or not to add ordered solvent (if the resolution is sufficient), automatic detection or adjustment of user-provided NCS selections, determining the set of atoms that should have their occupancies refined and automatic determination of occupancy constraints for atoms in alternative conformations. When joint refinement is performed using both X-ray and neutron data (Coppens *et al.*, 1981 ▶; Wlodawer & Hendrickson, 1981 ▶, 1982 ▶; Adams *et al.*, 2009 ▶; Afonine, Mustyakimov *et al.*, 2010 ▶), it is important to ensure that the cross-validation reflections are consistent between data sets. This check is performed automatically. If a mismatch is detected, *phenix.refine* will terminate and offer to generate a new set of flags consistent with both data sets.

The large set of configurable refinement parameters is presented to the user in a novel hierarchical organization, libtbx.phil, specifically designed to be user-friendly (Grosse-Kunstleve *et al.*, 2005 ▶). This is achieved *via* a simple syntax with the option to easily override selected parameters from the command line. This parameter-handling framework is completely general and can be reused for other purposes unrelated to refinement. A comprehensive and intuitive graphical user interface (GUI) built around this framework is also available, allowing users of all skill levels to use *phenix.refine*.

### 2.2. The main body of refinement: the refinement macro-cycle   

A refinement protocol typically consists of several steps, in which each step aims to optimize specific model parameters using dedicated methods. This is because of the following.

(i) The target function typically has many local minima. The objective of refinement is to approach the deepest minimum as closely as possible. A gradient-driven minimization can reach only the nearest local minimum; therefore, sophisticated search algorithms such as rotamer optimization (recently implemented in *phenix.refine*) or simulated annealing (Brünger *et al.*, 1987 ▶; Adams *et al.*, 1997 ▶; Brunger *et al.*, 2001 ▶; Brunger & Adams, 2002 ▶) may need to be applied.
(ii) Some groups of model parameters are highly correlated, *e.g.* isotropic displacement parameters and the exponential component of the overall scale-factor correction, ADPs and occupancies (Cheetham *et al.*, 1992 ▶), rigid-body ADPs modeled through TLS (for a review, see Urzhumtsev *et al.*, 2011 ▶), local atomic vibrations, and anisotropic scale and bulk-solvent parameters, *k*_sol_ and *B*_sol_ (Tronrud, 1997 ▶; Fokine & Urzhumtsev, 2002 ▶).
(iii) Different minimization methods imply different convergence radii for different model parameters (such as, for example, coordinates and ADPs) or for the same kind of parameters that have a large spread in magnitude (Agarwal, 1978 ▶; Tronrud, 1994 ▶).
(iv) As the model improves during refinement, a different model parameterization may be more appropriate. If additional model features become visible in the difference maps, such as new water molecules or ions, they may need to be reflected by additions or changes to the model. Further, erroneously modeled waters from earlier steps may need to be removed after a few macro-cycles since their ADPs and/or distances to other molecules may refine to implausible values.

The refinement protocol therefore consists of multiple steps repeated iteratively, in which each step is specifically tailored to the refinement of particular parameters. The required number of such steps depends on the data quality and initial model quality. Convergence of the particular refinement run is reached if the optimization of the model parameters does not lead to a significant improvement in the monitored criteria (refinement target function and *R* factors, for example). This section reviews the refinement steps.

#### 2.2.1. Total model structure factor, bulk-solvent correction, scaling and twin-fraction refinement   

The total model structure factor comprises a number of contributions, 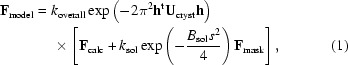
where *k*_overall_ is an overall scale factor, **U**_cryst_ is the overall anisotropic scale matrix (Sheriff & Hendrickson, 1987 ▶; Grosse-Kunstleve & Adams, 2002 ▶), **h** is a column vector with the Miller indices of a reflection and **h** ^t^ is its transpose, **F**_calc_ are the structure factors computed from the atomic model, *k*_sol_ and *B*_sol_ are flat bulk-solvent model parameters (Phillips, 1980 ▶; Jiang & Brünger, 1994 ▶), *s* ^2^ = **h** ^t^ **G** ^*^ **h**, where **G** ^*^ is the reciprocal-space metric tensor, and **F**_mask_ are structure factors calculated from a solvent mask (a binary function with zero values in the protein region and non-zeros values in the solvent region). The mask is computed using memory-efficient exact asymmetric units described in Grosse-Kunstleve *et al.* (2011 ▶). The mask-calculation parameters, *r*_solvent_ and *r*_shrink_, can be optimized in each refinement macro-cycle.

The structure factors from the atomic model, **F**_calc_, are computed using either fast Fourier transformation (FFT) or direct-summation algorithms (for a review, see Afonine & Urzhumtsev, 2004 ▶). Various X-ray and neutron scattering dictionaries are available (Neutron News, 1992 ▶; Maslen *et al.*, 1992 ▶; Waasmaier & Kirfel, 1995 ▶; Grosse-Kunstleve, Sauter *et al.*, 2004 ▶).

*phenix.refine* uses a very efficient and robust algorithm for finding the best values for *k*_sol_, *B*_sol_ and **U**_cryst_. The details of the algorithm, as well as a comprehensive set of references to relevant works, have been described previously (Afonine *et al.*, 2005*b* ▶). A radial-shell bulk-solvent model (Jiang & Brünger, 1994 ▶) is also available. In the case of refinement against twinned data, the total model structure factor is defined as
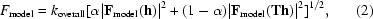
where α is a twin fraction and is determined by minimizing the *R* factor using a simple grid search in the [0, 0.5] range with a step of 0.01 and the matrix **T** defines the twin operator.

#### 2.2.2. Ordered solvent (water) modeling   

**Figure 1:**
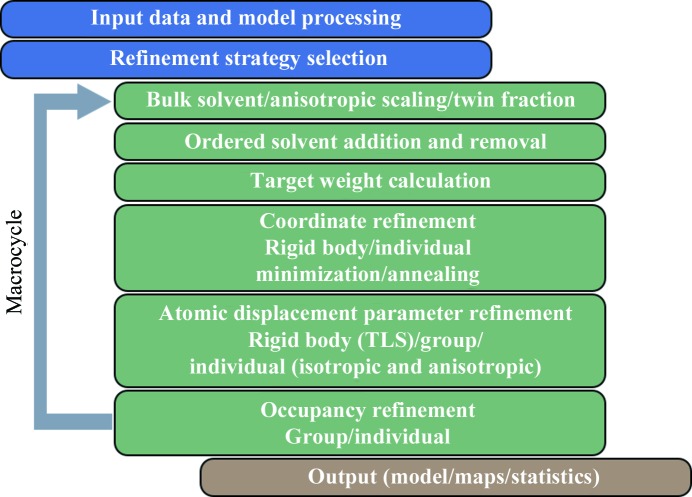
Flowchart of structure refinement as implemented in *phenix.refine.* The execution of some steps is subject to user-defined options. The main refinement body (shown with the gray arrow) is called a macro-cycle and is repeated several times. See text for details.

An automated protocol for updating the ordered solvent model can be applied during the refinement process. If requested by the user, waters are updated (added, removed and refined) in each macro-cycle as indicated in Fig. 1 ▶. Updating the ordered solvent model involves the following steps.

(i) Elimination of waters present in the initial model based on user-defined cutoff criteria for ADP, occupancy and inter­atomic distances (water–water and macromolecule–water), 2*mF*_obs_ − *DF*_model_ (see §[Sec sec2.3.1]2.3.1 for details) map values at water oxygen centers and map correlation coefficient values computed for each water O atom.
(ii) Location of new peaks in the *mF*_obs_ − *DF*_model_ map, followed by filtering of these peaks by their height and distance to other atoms. The filtered peaks are treated as new water O atoms with isotropic or anisotropic ADPs as specified by the user.
(iii) Depending on the refinement strategy (typically at high resolution), occupancies and individual isotropic or anisotropic ADPs of newly added water molecules can be refined prior to the refinement of all other parameters. This step is important because the newly placed waters have only approximate ADP values (which is usually the average *B* calculated from the structure). If a large number of new waters are added at once this may significantly increase the *R* factors at this step and have an impact on convergence of the refinement. In our experience, this effect is most pronounced for high-resolution data.
(iv) Unlike macromolecular atoms that are connected to each other *via* geometry restraints, the electron density is typically the only term in the target function keeping the O atom of a water molecule in place and occasionally it may happen that a density peak is insufficiently strong to keep a water molecule from drifting away during refinement. Therefore, in *phenix.refine* the water O-atom positions are analyzed with respect to the local density peaks and water molecules are automatically re-centered if necessary.
(v) For refinement using neutron or ultrahigh-resolution X-­ray data, water H or D atoms can be automatically located in the *mF*_obs_ − *DF*_model_ map and added to the model.

#### 2.2.3. Refinement targets and target weights   

Model parameters, such as coordinates and ADPs, are not refined simultaneously but at separate steps (see §[Sec sec2.2]2.2 for details). *phenix.refine* uses the following refinement target function for restrained refinement of individual coordinates,
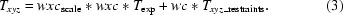
A similar function is used in restrained ADP refinement,
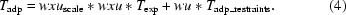
Here, *T*_exp_ is the crystallographic term that relates the experimental data to the model structure factors. It can be a least-squares target (LS; for example, as defined in Afonine *et al.*, 2005*a* ▶), an amplitude-based maximum-likelihood target (ML; for example, as defined in Afonine *et al.*, 2005*a* ▶) or a phased maximum-likelihood target (MLHL; Pannu *et al.*, 1998 ▶). For refinement of coordinates, *T*_exp_ can also be defined in real space (see below).

*T*_*xyz*_restraints_ and *T*_adp_restraints_ are restraint terms that introduce *a priori* knowledge, thus helping to compensate for the insufficient amount of experimental data owing to finite resolution or incompleteness of the data set typically observed in macromolecular crystallography. Note that the restraint terms are not used in certain situations, for example rigid-body coordinate refinement, TLS refinement, occupancy refinement, *f*′/*f*′′ refinement or if the data-to-parameter ratio is extremely high. In these cases the total refinement target is reduced to *T*_exp_.

The weights *wxc*_scale_, *wxc* and *wc* (or *wxu*_scale_, *wxu* and *wu*, correspondingly) are used to balance the relative contributions of experimental and restraints terms. The automatic weight-estimation procedure is implemented as described in Brünger *et al.* (1989 ▶) and Adams *et al.* (1997 ▶) with some variations and is used by default to calculate *wxc* and *wxu*. The long-term experience of using a similar scheme in *CNS* and *PHENIX* indicates that it is typically robust and provides a good estimate of weights in most cases, especially at medium to high resolution. In cases where this procedure fails to produce optimal weights, a more time-intensive automatic weight-optimization procedure may be used, as originally described by Brünger (1992 ▶) and further adopted by Afonine *et al.* (2011 ▶), in which an array of *wxc*_scale_ or *wxu*_scale_ values is systematically tested in order to find the value that minimizes *R*_free_ while keeping the overall model geometry deviations from ideality within a predefined range. The weight *wc* (or *wu*, correspondingly) is used to scale the restraints contribution, mostly duplicating the function of *wxc*_scale_ (or *wxu*_scale_), while allowing an important unique option of excluding the restraints if necessary (for example, at subatomic resolution). Setting *wc* = 0 (or *wu* = 0) reduces the total refinement target to *T*_exp_.

In maximum-likelihood (ML)-based refinement (Pannu & Read, 1996 ▶; Bricogne & Irwin, 1996 ▶; Murshudov *et al.*, 1997 ▶; Adams *et al.*, 1997 ▶; Pannu *et al.*, 1998 ▶) the calculation of the ML target (Lunin & Urzhumtsev, 1984 ▶; Read, 1986 ▶, 1990 ▶; Lunin & Skovoroda, 1995 ▶) requires an estimation of model error parameters, which depend on the current atomic parameters and bulk-solvent model and scales. Since the atomic parameters and the bulk-solvent model are updated during refinement, the ML error model has to be updated correspondingly, as described in Lunin & Skovoroda (1995 ▶), Urzhumtsev *et al.* (1996 ▶) and Afonine *et al.* (2005*a* ▶).

#### 2.2.4. Refinement of coordinates   

Depending on the resolution (or more formally the data-to-parameter ratio; Urzhumtsev *et al.*, 2009 ▶) and initial model quality, there are four main options for refinement of coordinates in *phenix.refine*: individual unrestrained (at subatomic resolution), individual restrained, constrained rigid-groups (also known as torsion-angle) or pure rigid-body refinement. Restrained individual coordinate refinement can be performed in real and/or reciprocal space. Coordinate refinement is performed using L-BFGS minimization (Liu & Nocedal, 1989 ▶) of the target *T*_*xyz*_ (2) with respect to atomic positional parameters (individual coordinates or rotation–translation parameters of rigid bodies or torsion-angle space variables), while keeping all other parameters fixed. Simulated annealing (SA) is an alternative option for optimizing the target *T*_*xyz*_ (2) and is known to be a powerful tool for escaping from local minima and therefore increasing the convergence radius of refinement (Brünger *et al.*, 1987 ▶). This option is available and can be used depending on the model and data quality, as well as the stage of refinement. SA can be performed in Cartesian or torsion-angle space (Grosse-Kunstleve *et al.*, 2009 ▶).

A highly optimized protocol for pure rigid-body refinement is available (the MZ protocol), in which the refinement begins with the lowest resolution zone using a few hundred low-resolution reflections and gradually proceeds to higher resolution by adding an optimal number of high-resolution reflections in each step (Afonine *et al.*, 2009 ▶). All of the parameters of this protocol have been selected to achieve the largest convergence radius with a minimal runtime. The algorithm does not require a user to truncate the high-resolution limits at *ad hoc* values.

Real-space refinement (RSR) of coordinates has a long history (Diamond, 1971 ▶; Deisenhofer *et al.*, 1985 ▶; Urzhumtsev, Lunin & Vernoslova, 1989 ▶; Jones *et al.*, 1991 ▶; Oldfield, 2001 ▶; Chapman, 1995 ▶; see also the discussion of and references to earlier original works in Murshudov *et al.*, 1997 ▶; Korostelev *et al.*, 2002 ▶). It is complementary to the more routinely used structure-factor-based reciprocal-space refinement. RSR optimizes the fit of the atoms to the current electron-density map. In *phenix.refine* the map is computed only once per macro-cycle. An RSR iteration is therefore typically much faster than a reciprocal-space refinement iteration and it is significantly more practical to systematically determine the optimal RSR relative weighting of *T*_exp_ and *T*_*xyz*_restraints_ in (3) compared with the reciprocal-space refinement weight optimization outlined in §[Sec sec2.2.3]2.2.3. The RSR weight determination in *phenix.refine* aims to find the largest weight for *T*_exp_ that still produces reasonable geometry. The current model is refined independently multiple times, each time using a different trial weight from an empirically determined range. The resulting geometry is evaluated by computing the maximum and average deviation of the model bond distances from ideal bond distances. Typically, the RSR procedure increases the *R* factors (work and free) for well refined structures, but for resolutions better than 3 Å we often observe important local corrections that are beyond the reach of SA (see §[Sec sec3]3). In such cases, subsequent reciprocal-space refinement usually leads to lower *R* factors than before RSR. In cases where the *R* factors increase beyond a user-definable threshold the RSR result is automatically discarded.

#### 2.2.5. Refinement of atomic displacement parameters (ADP refinement)   

An atomic displacement parameter (ADP) or *B* factor is a superposition of a number of nested contributions (Dunitz & White, 1973 ▶; Prince & Finger, 1973 ▶; Sheriff & Hendrickson, 1987 ▶; Winn *et al.*, 2001 ▶) that describe relatively small motions (within the validity of harmonic approximations), such as the following.

(i) Local atomic vibration.
(ii) Motion as part of a rotatable bond.
(iii) Residue movement as a whole.
(iv) Domain movement.
(v) Whole molecule movement.
(vi) Crystal lattice vibrations.

This parameterization can be made even more detailed (beyond the harmonic approximation; Johnson & Levy, 1974 ▶), but in practice most modern refinement programs use an approximation that consists of three main components (see, for example, Winn *et al.*, 2001 ▶), 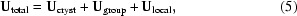
where **U**_total_ is the total atomic ADP.

**U**_cryst_ is a symmetric 3 × 3 matrix which models the common displacement of the crystal as a whole and some additional experimental anisotropic effects (Sheriff & Hendrickson, 1987 ▶; Usón *et al.*, 1999 ▶). This contribution is exactly the same for all atoms and thus it is possible to treat this effect directly while performing overall anisotropic scaling (Afonine *et al.*, 2005*a* ▶; see equation 1). **U**_cryst_ is forced to obey the crystal symmetry constraints. *phenix.refine* reports refined elements of the **U**_cryst_ matrix expressed on a Cartesian basis and uses the **B**_cart_ notation (Grosse-Kunstleve & Adams, 2002 ▶).

**U**_group_ is used to model the contribution to **U**_total_ arising from concerted motions of multiple atoms (group motions). It allows the combination of group motion at different levels (for example, whole molecule + chain + residue) and the use of models of different degrees of sophistication, such as general TLS, TLS for a fixed axis (a librational ADP; **U**_LIB_) and a simple group isotropic model with one single parameter. In its most general form, **U**_group_ can be **U**_TLS_ + **U**_LIB_ + **U**_subgroup_, where, for example, **U**_TLS_ would model the motion of the whole molecule or a large domain, **U**_subgroup_ would model the displacement of a smaller group such as a chain using a simpler one-parameter model and **U**_LIB_ would model a side-chain libration around a torsion bond using a simplified TLS model (Dunitz & White, 1973 ▶; Stuart & Phillips, 1985 ▶; currently, this approach is being implemented in *phenix.refine*). Depending on the current model and data quality, some components cannot be used: for example, **U**_group_ may be just **U**_TLS_.

If the TLS model is used then **U**_TLS_ = **T** + **ALA** ^t^ + **AS** + **S** ^t^ **A** ^t^ with 20 refinable **T** (translation), **L** (libration) and **S** (screw-rotation) matrix elements per group (Schomaker & Trueblood; 1968 ▶). The choice of TLS groups is often subjective and may be based on visual inspection of the molecule in an attempt to identify distinct and potentially independent fragments. A more rigorous and automated approach is implemented in the *TLSMD* algorithm (Painter & Merritt, 2006*a* ▶,*b* ▶). The *TLSMD* algorithm identifies TLS groups by splitting a whole molecule into smaller pieces followed by fitting of TLS parameters to the previously refined atomic *B* factors for each piece. Therefore, it is very important that the input ADPs for the *TLSMD* procedure are minimally biased by the restraints used in previous refinements and are meaningful in general (not reset to an arbitrary constant value, for example). In *PHENIX*, TLS groups can be determined fully automatically either as part of a refinement run or by using the *phenix.find_tls_groups* tool (Afonine, unpublished work).

Finally, small (in the harmonic approximation) local atomic vibrations, **U**_local_, can be modeled using a less detailed isotropic model that uses only one parameter per atom or using a more detailed (and accurate) anisotropic parameterization that includes six parameters per atom and therefore requires more experimental observations to be feasible. To enforce physical correctness of the refined ADPs, *phenix.refine* employs ADP restraints. In case of anisotropic ADPs these are simple similarity restraints (Schneider, 1996 ▶; Sheldrick & Schneider, 1997 ▶). For isotropic ADP refinement *phenix.refine* uses sphere ADP restraints first introduced by Afonine *et al.* (2005*b* ▶),
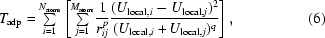
where *N*_atoms_ is the total number of atoms in the model, the inner sum spans over all *M*_atoms_ in the sphere of radius *R* around atom *i*, *r*_*ij*_ is the distance between two atoms *i* and *j*, **U**_local,*i*_ and **U**_local,*j*_ are the corresponding isotropic ADPs and *p* and *q* are empirical constants. By default, *R*, *p* and *q* are fixed at empirically derived values of 5.0 Å, 1.69 and 1.03, respectively, but they can also be changed by the user. The function reduces to a simple pair-wise similarity restraints target if *p* = *q* = 0 and the radius *R* is set to be approximately equal to the upper limit of a typical bond length.

The implementation of ADP refinement in *phenix.refine* is described in Afonine, Urzhumtsev *et al.* (2010 ▶) and Urzhumtsev *et al.* (2011 ▶).

#### 2.2.6. Occupancy refinement   

**Figure 2:**
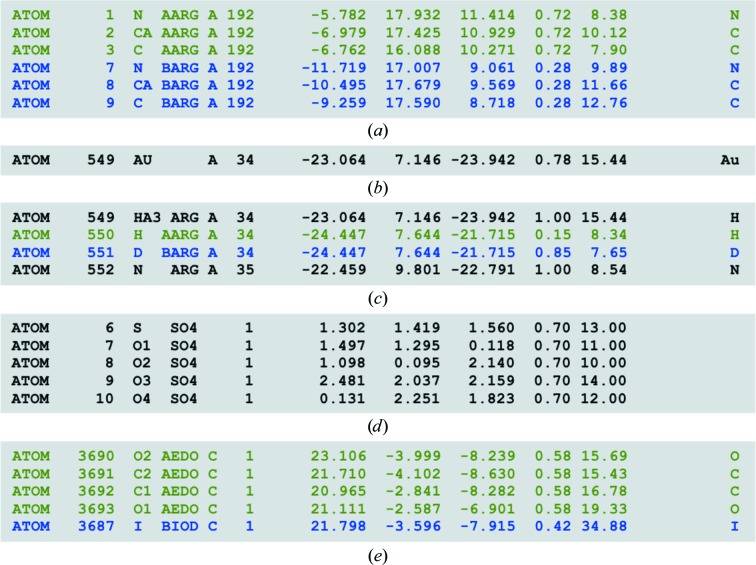
Illustration of typical scenarios for occupancy refinement that *phenix.refine* handles automatically. (*a*) Residue having several alternative conformations marked with altLoc identifiers (two in this example, A and B). It is essential that all conformers have identical chain identifiers and residue numbers, while residue names can be different as shown in example (*e*). All atoms within each conformer must have identical occupancies. The sum of occupancies over all conformers is constrained to 1. (*b*) Single atoms with occupancy not equal to 0 or 1. (*c*) Exchangeable H/D sites (used in refinement against neutron data collected from partially deuterated sample). (*d*) Single-residue molecule with identical occupancies for all atoms (but not equal to 1 or 0). A user can overwrite this behavior or/and define constraints for any number of selected atoms or groups of atoms.

Atomic occupancies can be used to model disorder beyond the harmonic approximation. With the default settings, *phenix.refine* always refines the occupancies of atoms in alternative conformations and those having partial nonzero occupancies at input (unless instructed otherwise by the user). The constraints for the occupancies of atoms in alternative conformations are constructed automatically based on the altLoc identifiers in the input PDB file. Also, a user can specify additional constraints on occupancies between any selected atoms. One can also perform a group occupancy refinement where one occupancy factor is refined per selected set of atoms and is constrained between predefined minimal and maximal values (0 and 1 by default). This can be useful for the refinement of partially occupied ligands, waters (when H or D are present) or other crystallization-solution components (Hendrickson, 1985 ▶). In the case of refinement of a partially deuterated structure against neutron data, the occupancies of exchangeable H/D sites are refined automatically and constraints are applied to ensure that the sum of related H and D occupancies is 1. *phenix.refine* does not currently build alternative conformations or H/D sites; external tools can be used for this, such as *phenix.ready_set* to add H/D atoms or *Coot* (Emsley & Cowtan, 2004 ▶; Emsley *et al.*, 2010 ▶) to add side chains in alternate conformations. Fig. 2 ▶ shows some typical situations that are addressed automatically by *phenix.refine*.

#### 2.2.7. Refinement of dispersive and anomalous coefficients (*f*′ and *f*′′)   

Given data with a significant anomalous signal, improved refinement results can be obtained by refining the coefficients *f*′ and *f*′′ of the anomalously scattering atoms (usually heavy atoms) and including them in the calculation of structure factors. Most commonly there is only one type of anomalous scatterer and it is reasonable to assume that the *f*′ and *f*′′ coefficients are identical for all anomalous scatterers of the same type in the asymmetric unit. In this case the data-to-­parameter ratio is very high and the refinement of the anomalous coefficients is very stable. Often it is possible to initiate refinement with *f*′ = 0 and *f*′′ = 0. For rare cases, *phenix.refine* also supports refinement of an arbitrary number of sets of *f*′ and *f*′′. Initial values may need to be specified in these cases.

### 2.3. Refinement output   

The following output is generated at the end of each *phenix.refine* run.

(i) A PDB file with the refined model and a summary of the refinement statistics in its header. The file header also contains ‘REMARK 3’ formatted records with refinement, model and data statistics, making it ready for PDB deposition.
(ii) A LOG file. A copy of the information that is printed to standard out during refinement. It contains the refinement statistics reported as the refinement progresses.
(iii) An MTZ file with four sections: (1) a copy of the input data (intensities or amplitudes) with associated error estimates (σs), *R*_free_ flags (if any) and Hendrickson–Lattman coefficients (if any); (2) data used in refinement (*F*_obs_ and corresponding σs); (3) total model structure factors **F**_model_ and (4) a number of Fourier map coefficients for the maps that can be visualized by the graphical program *Coot*. The data used in refinement may differ from the original input data as (*a*) the user can specify resolution and σ cutoffs, (*b*) *phenix.refine* performs outlier filtering and (*c*) if the input data are in the form of intensities *phenix.refine* will automatically convert them to amplitudes using the French and Wilson algorithm (French & Wilson, 1978 ▶).
(iv) A GEO file. This file lists all of the geometry restraints used in refinement, making it easy to inspect every restraint (type, ideal and current starting values where applicable) applied to an atom in question. Optionally, *phenix.refine* can also output a second GEO file that shows the value of each geometry restraint after refinement.
(v) An EFF file that contains all the parameters used in refinement run (this includes parameters specified in the command line, parameter file and default settings), and a DEF file with the parameters for a subsequent run.

#### 2.3.1. Map calculation and output   

In general, *phenix.refine* can output weighted p*mF_obs_ − q*DF_model_ and unweighted p*F_obs_ − q*F_model_ maps, where *p* and *q* can be any user-specified numbers. The phases used for computing these maps are either taken from the current model or the combination of model phases with the experimentally derived phases (if available). By default, *phenix.refine* outputs an MTZ file with several sets of Fourier map coefficients.

(i) Two 2*mF*_obs_ − *DF*_model_ maps, where one is computed using the *F*_obs_ used in refinement and the other is computed using manipulated *F*_obs_, where any missing *F*_obs_ are ‘filled’ in with *DF*_model_ (see below for details). To avoid any confusion, this is clearly indicated in the output MTZ file with map coefficients.
(ii) A difference *mF*_obs_ − *DF*_model_ map.
(iii) For anomalous data, if Bijvoet mates *F*_obs_(+) and *F*_obs_(−) are available, *phenix.refine* automatically outputs an anomalous difference map {[*F*_obs_(+) − *F*_obs_(−)]/2*i*}exp(*i*ϕ) computed with the model phase ϕ, where the imaginary unit *i* in the denominator introduces a −90° phase shift, (see, for example, Roach, 2003 ▶).

The coefficients *m* and *D* of likelihood-weighted maps (Read, 1986 ▶) are computed using the test set of reflections as described in Lunin & Skovoroda (1995 ▶) and Urzhumtsev *et al.* (1996 ▶). Other map types can also be output, such as average kick maps (AK maps; Guncar *et al.*, 2000 ▶; Turk, 2007 ▶; Pražnikar *et al.*, 2009 ▶) and *B*-factor sharpened maps (see Brunger *et al.*, 2009 ▶ and references therein) with the sharpening *B* factors determined automatically.

It is known that data incompleteness, especially systematic incompleteness (missing planes or cones of reciprocal space), can cause mild to severe map distortions (Lunin, 1988 ▶; Urzhumtsev, Lunin & Luzyanina, 1989 ▶; Lunin & Skovoroda, 1991 ▶; Tronrud, 1996 ▶; Lunina *et al.*, 2002 ▶; Urzhumtseva & Urzhumtsev, 2011 ▶). To compensate for data incompleteness, *phenix.refine* will ‘fill’ in missing observations with certain calculated values to reduce these map distortions. However, this procedure may introduce model bias and obviously the less complete the data, the higher the risk. By default, missing *F*_obs_ are ‘filled’ in with *DF*_model_ [similar to the procedure used in the *REFMAC* program (Murshudov *et al.*, 1997 ▶, 2011 ▶)], but there are other options possible, such as filling with 〈*F*_obs_〉, where the *F*_obs_ are averaged out in a resolution bin around the missing *F*_obs_, filling with simply *F*_model_ or even filling with random numbers generated around 〈*F*_obs_〉. Based on a limited number of tests, all of the above ‘filling’ schemes produce similar results, indicating the dominance of the phases rather than the amplitudes of the filled reflections. Clearly, this subject needs more systematic and thorough research (work in progress). However, one can effectively use both maps simultaneously, using the ‘filled’ map to help overcome difficult cases and using the unfilled map to confirm that map features have not been over-interpreted owing to model bias. For presentation purposes, it is recommended that unfilled maps be used so as to minimize any chance of misleading the viewer.

### 2.4. H atoms in refinement   

H atoms constitute about 50% of the atoms in a macromolecular structure, playing a crucial role in interatomic contacts (see, for example, Chen *et al.*, 2010 ▶ and references therein). H atoms also contribute to the atomic X-ray scattering (to **F**_model_). Information about H atoms (both, geometry and scattering) should therefore be used in refinement. In *phenix.refine* there are a number of tools that make handling of H atoms as easy and as automatic as possible at all resolutions and using any diffraction data source (X-ray, neutron or both simultaneously). A detailed overview of using H atoms in refinement can be found in Afonine, Mustyakimov *et al.* (2010 ▶).

### 2.5. Specific tools for refinement at subatomic resolution   

At subatomic resolution (see Urzhumtsev *et al.*, 2009 ▶ for a discussion of this definition), the residual electron-density maps begin to show some additional features that are not visible at lower resolutions, such as (i) density peaks for H atoms (for both macromolecule and water H atoms), (ii) electron-density peaks at interatomic bonds owing to bonding effects, (iii) lone-pair electrons and (iv) specific densities for ring-conjugated systems. The amount of these features visible in residual maps is a function of model quality and data resolution.

If a model is refined at ultrahigh resolution and the above features are not modeled, this model can be considered to be incomplete. It is well known that refining an incomplete model can have a negative effect on all model parameters: positional and *B* factors, for example (Lunin *et al.*, 2002 ▶; Afonine *et al.*, 2004 ▶). In addition, when refining a structure at such a high resolution one usually looks for very fine structural details (for example, Dauter *et al.*, 1995 ▶, 1997 ▶; Vrielink & Sampson, 2003 ▶; Petrova & Podjarny, 2004 ▶), which are often only seen as subtle features in residual maps close to the noise level. Completing the model is well known to improve the map quality (by reducing noise) and this is clearly demonstrated for the case of subatomic resolution residual maps (Afonine *et al.*, 2007 ▶; Volkov *et al.*, 2007 ▶).

*phenix.refine* possesses a number of tools specifically dedicated to model completion and refinement at subatomic resolution.

(i) Unrestrained coordinate and ADP refinement.
(ii) IAS model to address residual bonding density (Afonine *et al.*, 2007 ▶).
(iii) Individual or riding model for H atoms.
(iv) Automatic *mF*_obs_ − *DF*_model_ map-based location and optimization of water H atoms.
(v) Choice between FFT and direct-summation algorithms if the accuracy of the structure-factor calculation is of concern.

### 2.6. Specific tools for refinement at low resolution   

At low resolution (∼3.5 Å and worse), the electron-density map often provides little atomic detail and the traditional set of local restraints (bonds, angles, planarities, chiralities, dihedrals and nonbonded interactions) are insufficient to maintain known higher order structural organization (secondary structure) as well as other local geometry characteristics that are not directly restrained during refinement against higher resolution data (for example, peptide ϕ and ψ angles). At these low resolutions it is essential to include more *a priori* or external information in order to assure the overall correctness of the model. This information can be expressed through restraints to a known similar higher resolution (or homolologous) ‘reference’ structure (if available), to known secondary-structure elements or to target peptide ϕ and ψ angles in the Ramachandran plot. All these tools have recently been implemented in *phenix.refine* and details are discussed in this issue (Headd *et al.*, 2012 ▶).

Given low-resolution data, if there are several copies of a molecule in the asymmetric unit one can assume that these copies are essentially similar and therefore noncrystallographic symmetry (NCS) restraints can be applied to coordinates and ADPs (Hendrickson, 1985 ▶). This improves the data-to-parameter ratio at low resolution and therefore reduces the risk of overfitting (DeLaBarre & Brunger, 2006 ▶; for a practical example, see Braig *et al.*, 1995 ▶; it has been noted that nearly half of the low-resolution structures in the wwPDB contain NCS copies; see, for example, Kleywegt & Jones, 1995 ▶; Kleywegt, 1996 ▶).

In *phenix.refine* the coordinates and ADPs of NCS copies are harmonically restrained to the positions and ADPs of an average structure that is obtained by superposition and averaging of the NCS copies (Hendrickson, 1985 ▶). The NCS restraint term is added as an additional harmonic function to the geometry or ADP restraints terms. In ADP refinement the NCS restraints are only applied to **U**_local_ (Winn *et al.*, 2001 ▶; Afonine, Urzhumtsev *et al.*, 2010 ▶). Selections for NCS groups can either be provided by the user or they can be determined automatically. Currently, *phenix.refine* uses a simple algorithm for automatic NCS detection which is based on sequence alignment of the chains provided in the input PDB file. The automatically generated NCS groups should therefore be considered as a guide in generating a complete set of NCS restraints rather than as a best final answer.

If insufficient care is taken in defining the NCS groups, the above method may be counterproductive (Kleywegt & Jones, 1995 ▶; Kleywegt, 1996 ▶, 1999 ▶, 2001 ▶; Usón *et al.*, 1999 ▶). It is important not to use NCS restraints for truly variable fragments that are different between the NCS copies (certain side chains, flexible loops *etc.*), otherwise they will be forced to match the average structure, producing various local artifacts. An alternative approach restraining local interatomic distances has been published by Usón *et al.* (1999 ▶) and is used in *SHELXL* (Sheldrick, 2008 ▶). A similar approach using NCS restraints parameterized in torsion-angle space is available in *phenix.refine*.

### 2.7. GUI   

**Figure 3:**
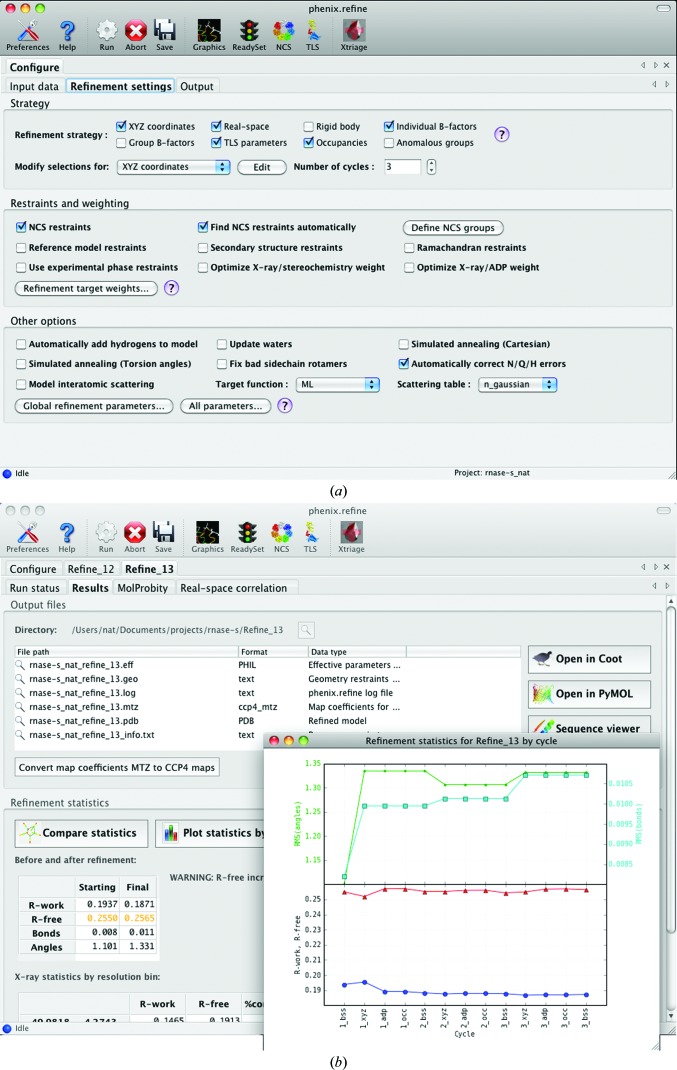
The graphical user interface (GUI) for *phenix.refine*. (*a*) Configuration tab showing the refinement strategy and commonly used restraint and optimization settings. (*b*) Display of results, including summary of output files, tables and graphs of statistics and links to molecular-graphics software.

The graphical interface for *phenix.refine* retains most of the functionality of the command-line program, with the same parameter template used to draw controls in the GUI (in many cases automatically). However, the arrangement and visibility of the controls have been tailored to minimize confusion for novice users, with only the most commonly used options displayed in the main window (Fig. 3 ▶ *a*). In the windows for individual protocols, advanced options are hidden by default, but may be toggled by a ‘user-level’ control. Several extensions in the GUI provide additional automation *via* links to other programs such as *phenix.ready_set*, *phenix.simple_ncs_from_pdb*, *phenix.find_tls_groups* and *phenix.xtriage*, all of which may be run interactively to generate parameters that are incorporated into the *phenix.refine* inputs. For parameters that define atom selections, a built-in graphical viewer allows dynamic visualization and modification of the selection. During and after refinement, progress is presented graphically as a plot showing the current *R* factors and geometry after each step. The final results (Fig. 3 ▶ *b*) include buttons to load the refined model and electron-density maps in *Coot* or *PyMOL* (DeLano, 2002 ▶). A comprehensive suite of validation tools largely derived from *MolProbity* (Davis *et al.*, 2007 ▶; Chen *et al.*, 2010 ▶) is run as the final step of refinement and these analyses are integrated into the display of results.

## 3. Selected examples   

In this section, we illustrate the application of *phenix.refine* to a broad range of refinement cases (Table 1 ▶). Standard protocols were used as dictated by the resolution of the diffraction data and the model characteristics. The refinement protocols were not manually optimized to produce the lowest free *R* factors.

### 3.1. Low-resolution structures   

The structures with PDB entries 1jl4 (Wang *et al.*, 2001 ▶), 2gsz (Satyshur *et al.*, 2007 ▶), 1yi5 (Bourne *et al.*, 2005 ▶), 2wjx (Clayton *et al.*, 2009 ▶), 3eob (Li *et al.*, 2009 ▶), 1av1 (Brouillette & Ananthara­maiah, 1995 ▶), 3bbw (Qiu *et al.*, 2008 ▶) and 2i07 (Janssen *et al.*, 2006 ▶) were selected because their published *R* factors are much higher than expected (Urzhumtseva *et al.*, 2009 ▶). We were interested to test whether it was possible to improve their refinement using *phenix.refine* in a straightforward fashion. Since all of these structures are reported at low resolution (4 Å or lower) the *phenix.refine* refinement included NCS (where available), secondary-structure and Ramachandran plot restraints for refinement of coordinates and a restrained isotropic model for the refinement of atomic displacement parameters. A bulk-solvent mask optimization was also performed (Brunger, 2007 ▶; DeLaBarre & Brunger, 2006 ▶). In all cases the *R* factors (both free and work) were reduced significantly and in two of them overlooked twinning was a likely cause of the unusually high published *R* factors. For structure 3bbw twinning was detected by *phenix.xtriage* and the corresponding twin operator was used in refinement.

### 3.2. Impact of ADP refinement   

The re-refinement of a synaptotagmin structure at 3.2 Å resolution (PDB entry 1dqv; Sutton *et al.*, 1999 ▶) emphasizes the importance of using a TLS parameterization not only as a way to reduce the number of refined parameters but more importantly to provide a more reasonable model for global domain motions (Urzhumtsev *et al.*, 2011 ▶). Restrained refinement of individual ADPs in *phenix.refine* reduces the published *R*_work_/*R*_free_ from 29.3/34.8% to 25.5/29.3%. Further combined refinement of TLS parameters and individual ADPs reduced *R*_work_/*R*_free_ to 22.5/25.5%.

### 3.3. High-resolution refinement   

**Figure 4:**
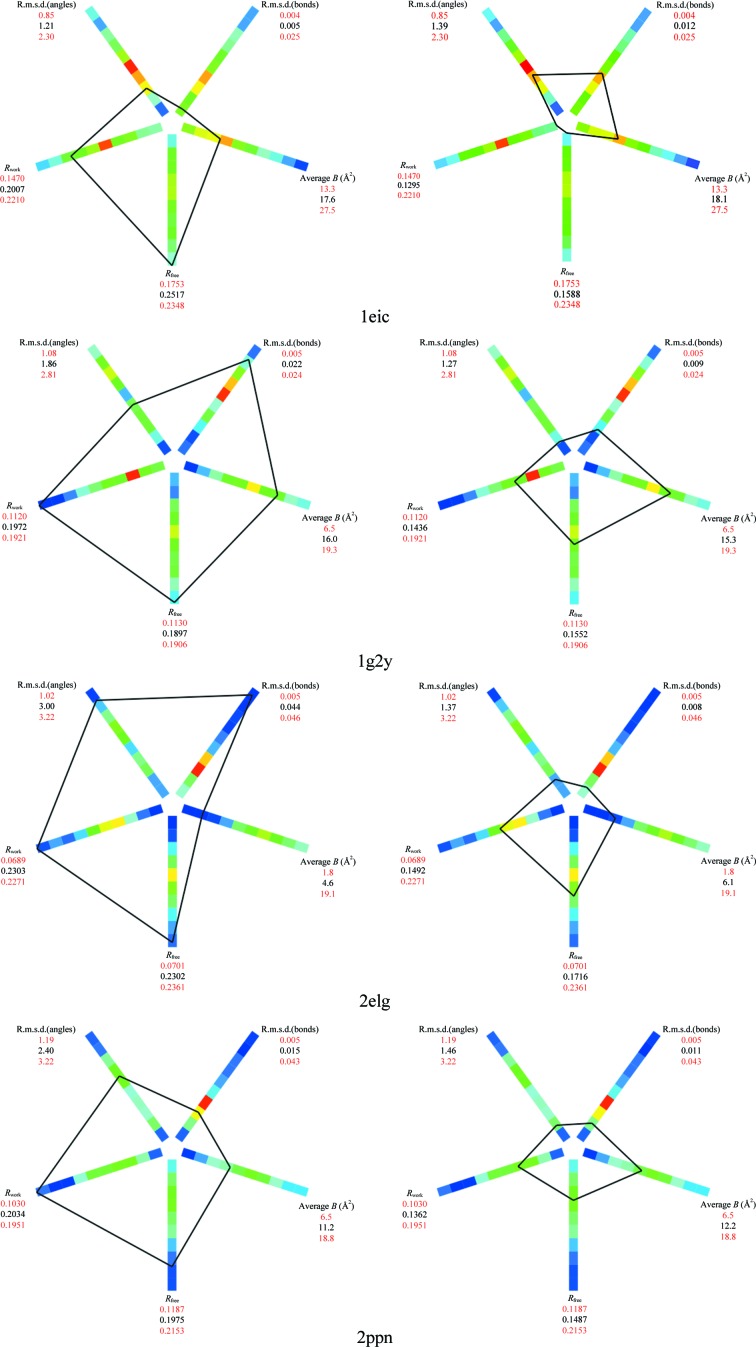
Polygon images (Urzhumtseva *et al.*, 2009 ▶) before (left) and after (right) re-refinement in *phenix.refine* for structures 1eic, 1g2y, 2elg and 2ppn. In all cases the polygon computed for structures before re-refinement in *phenix.refine* indicates one or more problems, for example high *R*_free_ and *R*_work_ and too small bond r.m.s.d. for 1eic or very high *R* factors and geometry deviations for 2elg (vertices are on the furthermost end of the histogram bar). Re-refinement in *phenix.refine* resulted in polygon vertices moved towards the center (squeezing the polygon) in most cases, indicating improvement of the corresponding model characteristics.

Given the relatively high resolution of 1.4 Å, the structure 1eic (Chatani *et al.*, 2002 ▶) has surprisingly high values of *R*_free_ and *R*_work_, as well as unusually small bond and angle deviations from ideal values (Fig. 4 ▶). Re-refinement with all anisotropic ADPs, automatic water update, target-weight optimization and added riding H atoms significantly improved these statistics. Other structures, 2elg (Ohishi *et al.*, 2007 ▶), 1g2y (Rose *et al.*, 2000 ▶) and 2ppn (Szep *et al.*, 2009 ▶), were also selected on the basis of unusually high *R* factors. Re-refining the models with added riding H atoms, anisotropic ADPs for all atoms except H atoms and automated water update resulted in a significant improvement in *R* factor and other statistics as illustrated by polygon images (Urzhumtseva *et al.*, 2009 ▶; Fig. 4 ▶).

### 3.4. Refinement against neutron data at medium and ultrahigh resolution   

**Figure 5:**
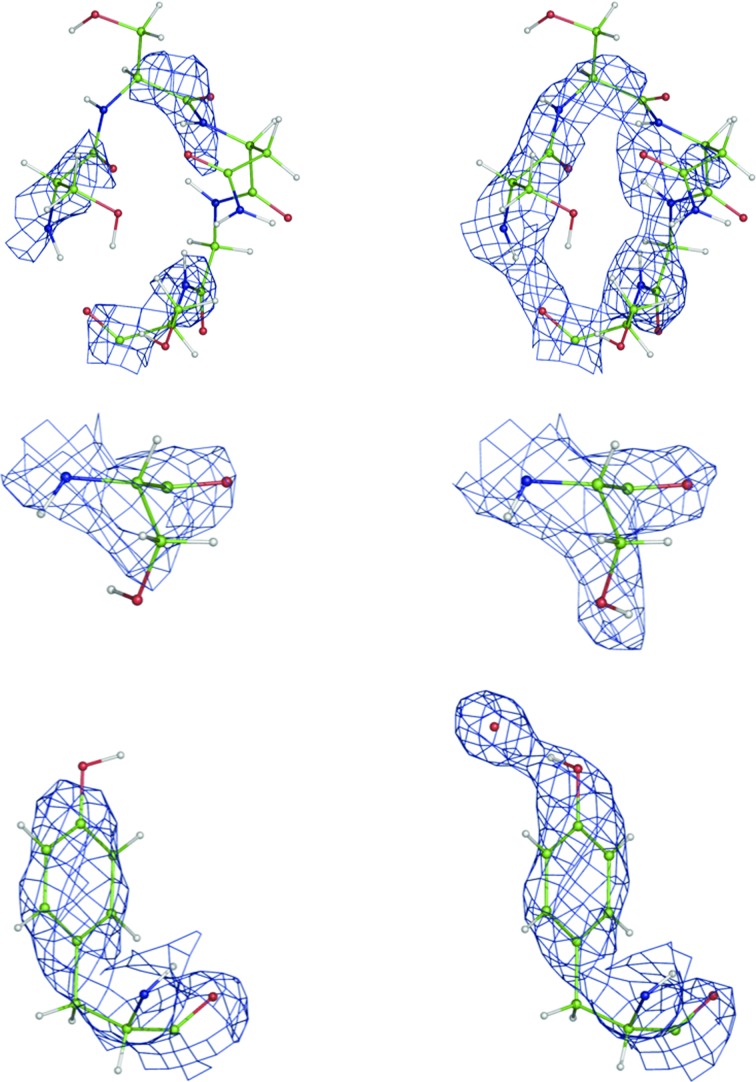
Selected examples of (2*mF*_obs_ − *DF*_model_, ϕ_model_) nuclear density map improvement after re-refinement of structure 1c57 (neutron data). Left, original structure; right, after re-refinement in *phenix.refine*. Maps are contoured at the 1.5σ level. Note the improved orientation of exchangeable H/D atoms at Ser and Tyr O atoms. The systematic lack of density around H atoms is a consequence of the negative scattering length of H atoms and related density-cancellation effects (Afonine, Mustyakimov *et al.*, 2010 ▶).

The structure 1c57 (Habash *et al.*, 2000 ▶) was obtained from a partially deuterated sample at 2.4 Å resolution. However, the PDB model does not contain any D atoms, resulting in the recalculated *R*_work_ of 30.0% and *R*_free_ of 33.9% being higher than the published values (27.0% and 30.1%, respectively). Automated rebuilding of H and H/D exchangeable atoms using *phenix.ready_set* followed by refinement in *phenix.refine* yielded significantly improved *R*_work_ and *R*_free_ factors of 20.4% and 25.7%, respectively (Table 1 ▶). The overall map improvement is also clear (Fig. 5 ▶ *a*). A number of rotatable H/D sites were reoriented into improved nuclear density by local real-space optimization (Figs. 5 ▶ *b* and 5 ▶ *c*). As another example, the availability of subatomic resolution data (0.65 Å) for the ur0013 structure (Guillot *et al.*, 2001 ▶) allowed partially unrestrained positional and all-atom anisotropic ADP refinement (including H atoms).

### 3.5. Combined real- and reciprocal-space refinement (dual-space refinement)   

**Figure 6:**
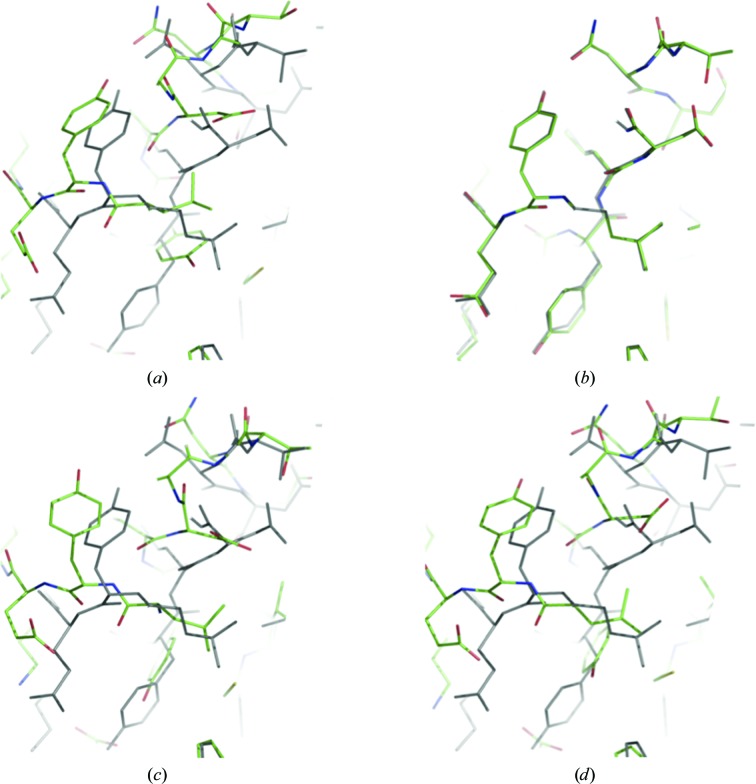
Structures after refinement of a severely distorted model, shown in (*a*), using different refinement protocols: (*b*) dual-space refinement, (*c*) refinement using minimization only and (*d*) combined refinement using minimization and simulated annealing. The best available refined model is shown in gray in all panels.

To illustrate the power of the dual-space refinement protocol implemented in *phenix.refine*, we selected a structure from the PDB (PDB entry 1txj; Vedadi *et al.*, 2007 ▶) and moved atoms in a such a way that the amount of introduced distortion is likely to put it beyond the convergence radius of traditional reciprocal-space minimization-based refinement. The model distortions included (i) switching to a different rotamer for each residue side chain; (ii) randomly moving (shaking using *phenix.pdbtools*) all coordinates with an r.m.s. coordinate shift of 1 Å followed by geometry regularization (also using *phenix.pdbtools*); (iii) removing all solvent and (iv) resetting all ADPs to the average value computed across all atoms. This resulted in an overall coordinate distortion r.m.s.d. of about 2.1 Å (Fig. 6 ▶ *a*) and an increase of the best available *R*_work_/*R*_free_ from 18.7/21.2% to 53.2/54.4%. Subsequently, we performed three independent refinement runs, each starting from the same distorted model. All refinements included ten macro-cycles of coordinate and isotropic ADP refinement combined with ordered solvent (water) updates. Coordinates in the first refinement were refined using L-BFGS minimization only. The second refinement included L-BFGS minimization and Cartesian simulated annealing performed during the first five macro-cycles. Finally, the third refinement was similar to the second one but included overall real-space refinement and local torsion-angle grid-search real-space correction of residues to best fit the density map and match the closest plausible rotameric state. The *R*_work_ and *R*_free_ after the three refinements were 46.1/52.2%, 41.5/48.8% and 20.8/23.7%, respectively. The refined models are shown in Figs. 6 ▶(*b*), 6 ▶(*c*) and 6 ▶(*d*). Clearly, the new dual-space refinement protocol was able to bring the distorted model back close to the best available refined model, while both simple minimization and combined minimization and simulated annealing failed to do so.

### 3.6. Including H atoms in refinement   

To illustrate the contribution of H atoms to refinement, we selected a structure from the PDB (PDB entry 3aci; Tsukimoto *et al.*, 2010 ▶) which was refined at 1.6 Å resolution to *R*_work_ = 14.1 and *R*_free_ = 18.8%. This structure was then refined with and without H atoms. Both refinement runs included three macro-cycles of positional and isotropic ADP refinement, automated water update and X-ray/restraints target-weight optimization. The refinement without H atoms yielded *R*_work_ = 14.6 and *R*_free_ = 18.3%. Refinement with H atoms resulted in *R*_work_ = 13.7 and *R*_free_ = 16.5%. We suggest that it is prudent to preserve the H atoms in the final model (and to record them in the PDB deposition file), as omitting them increases the *R*_work_ and *R*_free_ to 15.1% and 17.8%, respectively.

## 4. Remark regarding uncertainties in refinement results   

**Figure 7:**
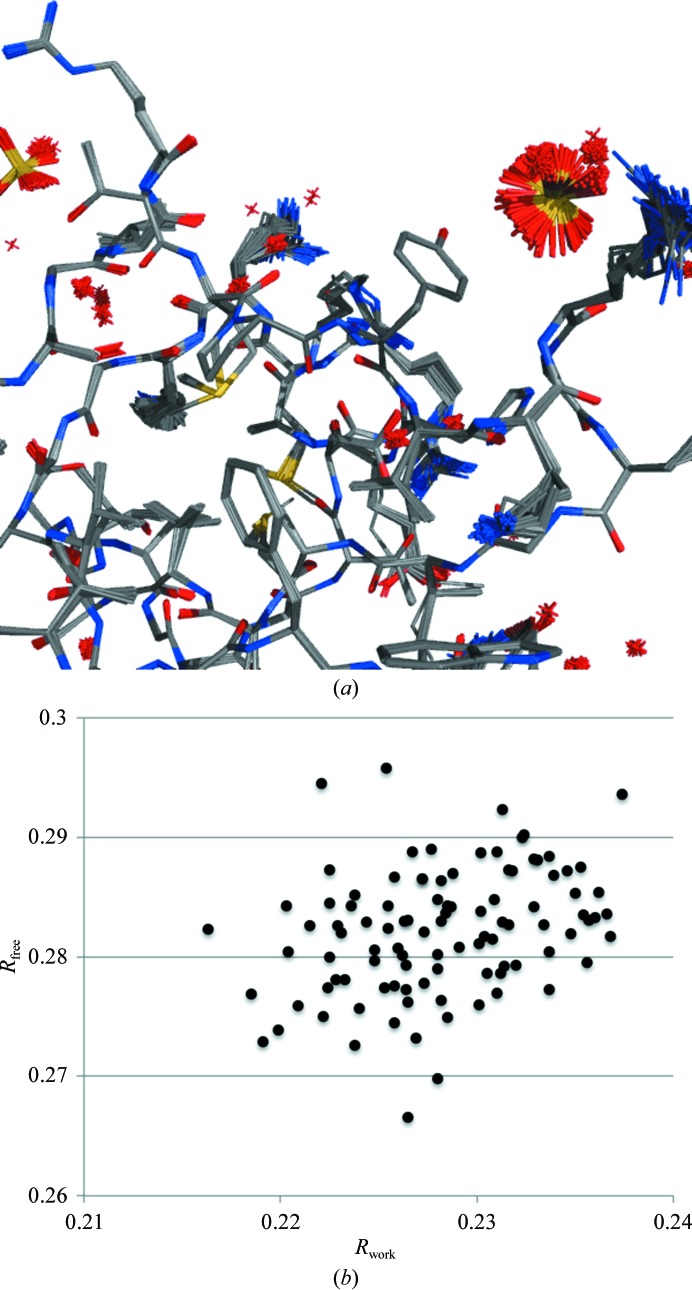
(*a*) Ensemble of structures illustrating the outcome of 100 identical simulated-annealing refinement runs apart from the random seed. (*b*) The distribution of *R*_work_ and *R*_free_ corresponding to each structure of the ensemble.

Given that the landscape of a macromolecular crystallography refinement target is very complex and the convergence radii of refinement protocols are generally very small in comparison, the outcome of a refinement run may strongly depend on the initial model and algorithmic parameters in ways that at first sight may not seem important. To illustrate this, we performed 100 identical SA refinement runs for a structure at 2 Å resolution, where the only difference between each refinement run was the random seed used to assign initial random velocities. The result is an ensemble of structures that are all similar in general but slightly different in detail (Fig. 7 ▶ *a*). The variation of structures within the ensemble reflects two phenomena: refinement artifacts (limited convergence radius and speed) and (probably to a lesser degree) structural variability (Terwilliger *et al.*, 2007 ▶). The spread of the ensemble broadens as the upper resolution limit becomes worse. The *R* factors also deviate further (Fig. 7 ▶ *b*). This variation is always important to keep in mind when comparing refinement results (*R* factors, for example) obtained with different refinement strategies or slightly different starting models.

## 5. Conclusions   

*phenix.refine* provides a comprehensive set of tools for refinement across a broad range of resolution limits (sub­atomic to low) using X-ray, neutron or both types of data simultaneously. A high degree of automation and robustness allows a range of refinement strategies to be used from a nearly ‘black box’-like default mode to the option of custom­izing more than 500 control parameters. All standard tools available for refinement using X-ray data are also available for refinement using neutron data. Any combination of available refinement strategies can be applied to any selected part of the structure. The GUI makes *phenix.refine* easy to use for both novice and experienced crystallographers.

The most recent developments include new or improved tools for refinement against low-resolution data (∼3.5 Å and lower), such as reference-model, secondary-structure and Ramachandran plot restraints, the latter being recommended in only the most challenging of circumstances such as very low resolution. NCS restraints parameterized in torsion-angle space will eliminate the need for subjective and often tedious selection of NCS groups. An improved target-weight optimization protocol is designed not only to yield a refined model with the best *R*_free_ but also to maintain the *R*_free_–*R*_work_ gap and model geometry within expected limits. A fast TLS group-determination algorithm allows fully automated assignment of TLS groups as part of the refinement run. Our initial results incorporating real-space methods into the refinement protocol (dual-space refinement) show a significant increase in the convergence radius of refinement that is not typically achievable using only reciprocal-space methods.

Future development plans include further improvements of the tools for low-resolution refinement, the expanded use of real-space methods for fast local model completion and rebuilding, the implementation of twinning-specific maximum-likelihood targets, methods for refinement of very incomplete atomic models, better modeling of local structural anisotropy and improving the bulk-solvent model to account for hydrophobic cores and alternative conformations. More automated decision-making will also be implemented for determining the optimal model parameterization and refinement strategy for different situations.

Finally, others have shown (Joosten *et al.*, 2009 ▶) that it is possible to apply modern refinement and model-rebuilding algorithms to improve structures deposited in major public databases such as the PDB. A number of examples in this manuscript illustrate that the application of methods in the *phenix.refine* program can potentially extend these improvements and lead to even better models.

The *PHENIX* software is available at http://www.phenix-online.org free of charge for academic users and through a consortium for commercial users.

## Figures and Tables

**Table 1:** *R* factors, Ramachandran plot outliers (RO) and *MolProbity* clashscores (CS; Davis *et al.*, 2007 ▶) for selected structures extracted from the PDB (published), extracted from *PDB_REDO* and after refinement using *phenix.refine* All data cutoffs (resolution, ) were applied as reported in the original works in order to maintain the same reflections used in the calculations.

Extracted from REMARK records of PDB file header				Calculated with *PHENIX* from original PDB†			*PDB_REDO* ‡			Re-refined in *phenix.refine* §		
Code¶	*d*_min_ ()	Data source	*R*_work_/*R*_free_ (%)	*R*_work_/*R*_free_ (%)	RO (%)	CS	*R*_work_/*R*_free_ (%)	RO (%)	CS	*R*_work_/*R*_free_ (%)	RO (%)	CS
1jl4	4.3	X-ray	42.0/45.3	34.2/37.0	0.74	40.0	23.9/31.4	0.74	25.8	24.2/30.9	0.92	13.3
2gsz	4.2	X-ray	34.3/40.6	31.0/36.1	1.33	37.1	34.3/39.9	1.33	37.1	25.8/32.7	0.98	25.7
1yi5	4.2	X-ray	33.1/37.8	30.3/33.9	2.79	17.6	24.1/31.1	2.86	15.9	25.9/28.5	0.98	15.5
2wjx	4.1	X-ray	29.0/35.4	33.7/37.0	0.28	24.5	33.8/36.7	0.28	24.6	28.5/31.9	0.28	11.8
1av1	4.0	X-ray	38.2/42.8	35.1/38.4	1.26	55.4	35.1/40.1	1.01	58.1	33.3/36.3	0.50	16.1
3bbw	4.0	X-ray	30.2/35.4	21.8/22.8	2.82	21.6	18.4/19.9	1.32	15.9	18.1/21.5	0.94	11.1
2i07	4.0	X-ray	27.3/32.3	26.8/28.7	2.04	24.9	22.5/28.5	1.84	20.4	21.5/26.4	0.79	14.6
3eob	3.6	X-ray	26.7/33.3	26.7/33.5	2.08	40.4	27.6/34.5	2.08	40.5	26.8/29.3	0.50	12.6
1dqv	3.2	X-ray	29.3/34.8	30.2/na††	10.3	93.1	na	na	na	22.2/25.5	0.37	24.8
1c57	2.4	Neutron	27.0/30.1	30.0/33.9	0	6.2	na	na	na	20.4/25.7	0	11.2
1jmc	2.4	X-ray	21.2/33.0	20.1/31.6	1.69	31.0	21.5/28.9	1.69	14.6	22.0/27.2	1.27	16.4
1eic	1.4	X-ray	20.1/25.4	20.1/25.2	0.82	21.7	16.0/17.7	0.82	11.9	12.9/15.9	0.82	20.0
2elg	1.0	X-ray	23.2/24.7	22.85/na	0	7.3	14.1/16.9	0	2.4	14.1/16.0	0	7.3
1g2y	1.0	X-ray	19.5/19.8	19.7/19.0	0.89	10.0	16.1/17.5	0.89	8.6	14.4/15.3	0.89	15.5
2ppn	0.92	X-ray	20.9/19.9	20.4/19.8	0	8.1	15.4/16.0	0	8.6	13.3/14.7	0	14.6
ur0013	0.65	Neutron	9.5/na	9.1/na	0	0	na	na	na	7.8/9.6	0	0

† Calculated in *PHENIX* after applying resolution and cutoffs, as reported in the PDB file header.

‡ *R* factors as reported on the *PDB_REDO* web site (Joosten *et al.*, 2009 ▶; http://www.cmbi.ru.nl/pdb_redo/); RO and CS computed using *PHENIX*.

§ Re-refinement of PDB-deposited structures using *phenix.refine*. Refinement strategy (model parameterization and number of refinement macro-cycles) varies depending on model and data quality. See text for details.

¶ PDB or NDB (Berman *et al.*, 1992 ▶) code.

†† na, value is not available either owing to a missing cross-validation set of reflections or the entry is not available in the database.
